# Cognitive predictors of adolescent social anxiety

**DOI:** 10.1016/j.brat.2020.103801

**Published:** 2021-02

**Authors:** Kenny Chiu, David M. Clark, Eleanor Leigh

**Affiliations:** a Department of Psychology, Institute of Psychiatry, Psychology & Neuroscience, King'sCollege London, London, UK; b Department of Experimental Psychology, University of Oxford, Oxford, UK

**Keywords:** Adolescent, Prospective, Social anxiety, Social cognitions, Safety behaviours, Self-focused attention, Pre-event processing, Post-event processing

## Abstract

**Background:**

Identifying psychological processes that maintain social anxiety holds promise for improving treatment outcomes for young people. Experimental and prospective studies in adults suggest negative social cognitions, safety behaviours, self-focused attention, and pre- and post-event processing are all implicated in the maintenance of social anxiety. Despite social anxiety typically starting in adolescence, prospective studies examining these cognitive processes in youth are lacking. The current study examined prospective associations between these five cognitive processes and social anxiety in a sample of 614 participants (53% girls; aged 11–14 years).

**Methods:**

Psychological processes, social anxiety symptoms, and depressive symptoms were assessed using self-report questionnaires at two time points.

**Results:**

Negative social cognitions, safety behaviours, self-focused attention, and post-event processing predicted prospective levels of social anxiety over and above the effect of baseline levels of social anxiety. When these process variables were entered together in a regression model, three of them were independently associated with prospective social anxiety. Neither pre- nor post-event processing independently predicted later social anxiety over and above the effects of other psychological process variables.

**Conclusions:**

The findings indicate that these psychological processes are promising targets for treatment in adolescent social anxiety.

## Highlights

• The applicability of the cognitive model of Clark & Wells was tested in a sample of 614 adolescents.
• Negative social cognitions independently predicted increases in social anxiety.
• Safety behaviours independently predicted increases in social anxiety.
• Self-focused attention independently predicted increases in social anxiety.
• Unexpectedly, neither pre- or post-event processing was an independent predictor of social anxiety.

Social Anxiety Disorder (SAD) is the most common anxiety disorder (Kessler et al., 2005), a predictor of later depression and suicidality (Stein et al., 2001), and associated with social, academic, and occupational impairment (Burstein et al., 2011; de Lijster et al., 2018). Nearly all cases begin before adulthood (Wittchen, Stein, & Kessler, 1999). Despite this, we know relatively little about the psychological processes implicated in the persistence of adolescent social anxiety. Building on research in adults, we test the hypothesis that prospective levels of adolescent social anxiety can be predicted by the following psychological processes: negative social cognitions, safety behaviours, self-focused attention, and pre- and post-event processing.

A number of cognitive and behavioural models have been developed to explain individual variability in *adult* social anxiety (for a review see Wong & Rapee, 2016), all emphasising the role of negative thoughts in social situations (e.g. “I will be unable to speak”, “They will think that I am boring”). Several accounts also highlight the role of other key psychological variables (Clark & Wells, 1995; Hofmann, 2007; Rapee & Heimberg, 1997). For example, the Clark and Wells’ (1995, pp. 69–93) model suggests certain psychological processes prevent disconfirmation of negative thoughts and maintain social anxiety. It is suggested that in social situations, socially anxious individuals will turn their attention inwards, monitoring themselves and how they are coming across. As a result, they fail to notice how others respond to them, and instead mistakenly use internal information, such as anxious feelings and negative images, as evidence for their negative beliefs (Hirsch, Meynen, & Clark, 2004; Mansell & Clark, 1999). Negative thoughts and images also motivate the use of safety behaviours, for example, speaking less or rehearsing sentences in mind. Although these behaviours are motivated by a desire to prevent or mitigate feared outcomes (Salkovskis, 1991), they inadvertenedly maintain negative beliefs, increase self-focus and anxiety, and may contaminate social interactions (Gray, Beierl, & Clark, 2019; McManus, Sacadura, & Clark, 2008). Socially anxious individuals commonly report worrying before and after social events, which intensifies anxiety and distress, and exaggerates negative thoughts and beliefs (Dannahy & Stopa, 2007; Hinrichsen & Clark, 2003).

Numerous studies, using concurrent and prospective correlational designs, and experimental manipulations, have provided strong support for the role of these processes in maintaining *adult* social anxiety (for a review see Wong & Rapee, 2016). But what do we know about the relevance of these processes to adolescents? Recently, Leigh and Clark (2018) reviewed studies examining the association between these processes and social anxiety in youth. There was a strong association between negative social cognitions and social anxiety. Safety behaviours, self-focused attention, and pre- and post-event processing were also associated with social anxiety. However, conclusions were limited because almost all studies included in the review were cross-sectional and correlational in nature. For example, Schreiber, Hofling, Stangier, Bohn, and Steil (2012) asked adolescents aged 14−20 years to complete self-report measures of psychological process variables and social anxiety symptoms. Negative social cognitions and safety behaviours, but not self-focused attention, pre- or post-event processing were associated with social anxiety. This study, and that of Hodson, Mcmanus, and Clark (2008), measured multiple psychological processes in one study, allowing examination of the unique contribution of each process in accounting for individual variability in social anxiety. However, we cannot determine from these studies whether the processes described are precedents of social anxiety. Prospective studies or experimental manipulations would provide a stronger test for causality as they meet the criterion of temporal precedence (i.e. the cause must precede the effect). Prospective observational studies, in particular, can help understand whether the associations between psychological processes and social anxiety are observable in a natural environment.

The aim of the present study was to examine whether the above mentioned processes are implicated in the maintenance of social anxiety in young people. Using a prospective study design, we measured psychological processes at baseline and monitored changes in social anxiety over time. It was hypothesised that, after controlling for baseline levels of social anxiety, negative social cognitions, safety behaviours, self-focused attention, and pre- and post-event processing would each be associated with later social anxiety (Hypothesis 1), and that each variable would be independently associated with later social anxiety (Hypothesis 2). Potential age difference in social anxiety was considered because the processes described may be underpinned by cognitive processes that are developing during adolescence (e.g. self-consciousness; Rankin, Lane, Gibbons, & Gerrard, 2004). Possible gender difference in social anxiety was examined because studies have found that females report higher levels of social anxiety than males (Asher & Aderka, 2018; Asher, Asnaani, & Aderka, 2017).

## 1 Methods

### 1.1 Participants and procedures

Participants in years 7–9 (aged 11–14 years) were recruited from two mainstream, non-selective, and state-funded secondary schools in London, UK. None had difficulty reading or understanding English. Participants aged 15 or above (*n* = 1) were excluded (see Supplementary Materials Fig. S.1). 13% of the pupils were eligible for free school meals, for 13% English was not their first language, and 12% of whom had special educational needs. These figures were consistent with national statistics (UK Statistics Authority, 2018). Six hundred fourteen participants attended a baseline assessment at Time 1 (T1) and completed a self-report questionnaire pack evaluating social anxiety symptoms, depression symptoms, and psychological processes. Four to six months later, 452 of them attended a follow-up assessment at Time 2 (T2) and completed a self-report questionnaire measuring social anxiety symptoms. There were no significant differences in age, gender, social anxiety, depression, and psychological processes between the two schools (*ps* > .05). In addition, there were no significant differences between participants with complete or missing data for social anxiety at follow-up (*ps* > .05). The study procedures were approved by the Central University Research Ethics Committee at the University of Oxford (Reference number: R54283/RE001). Written informed assent was obtained from all participants and opt-out consent was obtained from parents or carers.

### 1.2 Measures

Negative Social Cognitions—The Adolescent Social Cognitions Questionnaire (ASCQ) is a 22-item self-report scale measuring the frequency and belief of negative automatic thoughts in social situations, adapted from the Social Cognitions Questionnaire (SCQ; Wells, Stopa, & Clark, 1993). It includes items such as “I will be unable to speak” and “People will stare at me”. The SCQ has good internal consistency in an adolescent sample (Hodson et al., 2008). It has been used to measure treatment outcomes of adults with SAD (e.g. Stott et al., 2013). Mean scores were obtained for the frequency of negative automatic thoughts, ranging from 1 (*never*) to 5 (*always*). The internal consistency for the frequency subscale this sample was α = 0.96 (T1).

Safety Behaviours—The Adolescent Social Behaviour Questionnaire (ASBQ) is a 33-item self-report questionnaire measuring the frequency by which one behaves in certain ways to seek safety in social situations. It was adapted from the Social Behaviour Questionnaire (SBQ; Stopa & Clark, 1993) with four additional items following consultation with service users. Sample items included: “Get other people to speak for me or do things for me” and “Have an excuse or ‘get out’ planned”. Each item has a 0–3 rating represented by ‘*never*’, ‘*sometimes*’, ‘*often*’, and ‘*always*’. A summary score was derived from the average of all items. The SBQ has shown good internal consistency in adolescents (Schreiber et al., 2012) and good discriminant validity when compared to a measure of anxious appearance in adults (Makkar & Grisham, 2011). The internal consistency of the ASBQ in this sample was α = 0.92 (T1).

Self-focused Attention, Pre- and Post-event Processing—The Adolescent Social Phobia Weekly Summary Scale (ASPWSS) is a 6-item self-report visual analogue scale assessing different aspects of social anxiety on a 0–8 rating scale, adapted from the Social Phobia Weekly Summary Scale (SPWSS; Clark et al., 2003). Three items from the scale were used in the present study: ‘focus of attention in difficult social situations’ (item 4), ‘pre-event processing’ (item 5), and ‘post-event processing’ (item 6). Participants rated the extent to which they focused on themselves (from 0 to 8; ‘*entirely externally focused*’ to ‘*entirely self-focused*’). A Likert scale (from 0 to 8; ‘*not at all’* to ‘*always*’) was used to measure their tendency to worry before (pre-event processing) and after social situations (post-event processing). The 6-item scale has good internal consistency (Clark et al., 2003; Schreiber et al., 2012) and has been used as an outcome measure of treatment for adult SAD (Clark et al., 2006). The internal consistency of the 6-item scale in this sample was α = 0.74 (T1).

Social Anxiety—The Liebowitz Social Anxiety Scale for Children and Adolescents-Self Report version (LSAS-CA; Masia-Warner et al., 2003) is a 24-item self-report scale measuring social anxiety in young people aged between 7 and 18 years. This scale assesses levels of fear and avoidance in social and performance situations. Each item has a 0–3 rating represented by ‘*none*’, ‘*mild*’, ‘*moderate*’, and ‘*severe*’. The total score was obtained by adding up the 48 items on fear and avoidance. This scale has good reliability and validity (Masia-Warner et al., 2003). The internal consistency in this sample was α = 0.96 (T1) and α = 0.97 (T2).

Depression—The Short Mood and Feelings Questionnaire (SMFQ; Angold et al., 1995) is a 13-item self-report questionnaire assessing depressive symptoms in young people aged between 6 and 17 years. Each item ranges from 0 (*not true*) to 2 (*true*). Total scores were obtained by summing up all the items. This scale has good reliability and validity (Angold et al., 1995). The internal consistency in this sample was α = 0.91 (T1).

### 1.3 Data analysis plan

Statistical analyses were performed using R version 3.5.6 (R Core Team, 2019). Mahalanobis distance was used to detect multivariate outliers and they were removed from the dataset (*n* = 2). At item level, there was 15% of missing data. Little's MCAR (missing completely at random) test showed a non-significant result (*p* > .05), meaning that the data was MCAR. Mean substitution was performed when less than 5% of items were missing in each questionnaire. Questionnaires with more than 5% of missing items were treated as missing variables. At variable level, the non-significant result (*p* > .05) obtained from Little's MCAR test indicated the data was MCAR. We performed multiple imputation to account for missing data. The *mice* package (van Buuren & Groothuis-Oudshoorn, 2011) was used to perform multiple imputations, with 15 imputed datasets and 20 iterations. The analyses were repeated using participants with complete data (*n* = 351), and the results were consistent with results obtained from multiply imputed datasets (see Supplementary Materials Table S1 and Table S2).

Descriptive statistics and percentages of missing data are reported. A series of independent sample *t*-tests were conducted to examine if there were significant differences in study variables (i.e. age, social anxiety, depression, and psychological processes) between schools and genders. Shapiro-Wilk tests and inspection of skewness and kurtosis were performed. Descriptive statistics and correlations of imputed variables are presented. Multiple linear regression analyses were conducted to examine the two hypotheses.

## 2 Results

### 2.1 Descriptive statistics

Table 1presents the descriptive statistics, results of independent sample *t*-tests for comparing scores between boys and girls, and percentages of missing data. The final sample consisted of 614 participants (53% girls) aged 11–14 years (*M* = 12.97, *SD* = 0.87). Levels of baseline social anxiety (*M* = 41.01, *SD* = 28.73) and depression (*M* = 7.10, *SD* = 6.44) were comparable to findings of observational studies with young people (Abdollahi, Yaacob, Talib, & Ismail, 2015; McKenzie et al., 2011; Schmits, Maurage, Thirion, & Quertemont, 2014; Shachar, Aderka, & Gilboa-Schechtman, 2014). Girls reported higher levels of baseline social anxiety (*p* < .001) and depression (*p* < .001) than boys. They also reported significantly higher levels of negative social cognitions (*p* < .001), safety behaviours (*p* < .001), pre-event processing (*p* < .001), and post-event processing (*p* < .01) than boys. Girls' self-reported levels of self-focused attention (*p* = .42) were comparable to those reported by boys. Normality tests indicated all the study variables (i.e. age, social anxiety, depression, and psychological processes) were normally distributed. Table 2 presents the **descriptive statistics** and Pearson's correlations of imputed variables. All the psychological process variables were significantly and positively associated with levels of social anxiety. Age was positively associated with social anxiety, depression, negative social cognitions, safety behaviours, and pre- and post-event processing. Given these findings, the effects of age and gender were controlled for in subsequent regression analyses.

**Table 1:** Descriptive statistics, results of independent sample t-tests between boys and girls, and percentages of missing data.

Variable	Mean (SD)	Mean (SD)	Mean (SD)	*t*-test and *p*-value	Percentages of missing data
	All	Girls	Boys		
1. T1 Age	12.97 (0.87)	13.00 (0.89)	12.93 (0.86)	*t*(588) = 1.02, *p* = .31	4
2. T1 Social anxiety	41.01 (28.73)	47.04 (31.00)	33.90 (23.90)	*t*(599) = 5.74, *p* < .001***	2
3. T1 Negative social cognitions	2.05 (0.88)	2.31 (0.95)	1.75 (0.69)	*t*(514) = 7.51, *p* < .001***	16
4. T1 Safety behaviours	1.01 (0.46)	1.10 (0.49)	0.89 (0.45)	*t*(542) = 4.97, *p* < .001***	12
5. T1 Self-focused attention	3.90 (1.92)	3.96 (1.99)	3.83 (1.84)	*t*(579) = 0.80, *p* = .42	5
6. T1 Pre-event processing	4.29 (2.57)	4.73 (2.53)	3.78 (2.53)	*t*(586) = 4.53, *p* < .001***	4
7. T1 Post-event processing	4.30 (2.57)	4.60 (2.60)	3.97 (2.51)	*t*(584) = 2.98, *p* < .01**	5
8. T1 Depression	7.10 (6.44)	8.87 (7.07)	5.08 (4.92)	*t*(534) = 7.10, *p* < .001***	13
9. T2 Social anxiety	39.51 (29.74)	45.34 (31.00)	32.15 (26.30)	*t*(439) = 4.74, *p* < .001***	28

Note. *p < .05. **p < .01. ***p < .001. *SD* = standard deviation. Social Anxiety = Liebowitz Social Anxiety Scale for Children and Adolescents-Self Report total score; Safety behaviours = Adolescent Social Behaviour Questionnaire mean score; Negative social cognitions = Adolescent Social Cognitions Questionnaire frequency mean score; Self-focused attention = Adolescent Social Phobia Weekly Summary Scale item 4; Pre-event processing = Adolescent Social Phobia Weekly Summary Scale item 5; Post-event processing = Adolescent Social Phobia Weekly Summary Scale item 6; Depression = Short Mood and Feelings Questionnaire total score.

**Table 2:** Descriptive statistics and Pearson's correlations of study variables.

Variable	Mean (SD)	1	2	3	4	5	6	7	8
1. T1 Age	12.96 (0.87)								
2. T1 Social anxiety	40.83 (28.61)	.19**							
3. T1 Negative social cognitions	2.05 (0.88)	.16**	.72**						
4. T1 Safety behaviours	1.00 (0.48)	.13**	.70**	.78**					
5. T1 Self-focused attention	3.91 (1.93)	.01	.13**	.09**	.14**				
6. T1 Pre-event processing	4.30 (2.57)	.17**	.54**	.59**	.60**	.16**			
7. T1 Post-event processing	4.31 (2.57)	.27**	.48**	.55**	.58**	.15**	.58**		
8. T1 Depression	7.03 (6.37)	.17**	.59**	.76**	.63**	.09**	.50**	.45**	
9. T2 Social anxiety	38.63 (29.61)	.14**	.77**	.67**	.66**	.21**	.44**	.44**	.57**

*Note.* *p < .05. **p < .01. *SD* = standard deviation. Social Anxiety = Liebowitz Social Anxiety Scale for Children and Adolescents-Self Report total score; Negative social cognitions = Adolescent Social Cognitions Questionnaire frequency mean score; Safety behaviours = Adolescent Social Behaviour Questionnaire mean score; Self-focused attention = Adolescent Social Phobia Weekly Summary Scale item 4; Pre-event processing = Adolescent Social Phobia Weekly Summary Scale item 5; Post-event processing = Adolescent Social Phobia Weekly Summary Scale item 6; Depression = Short Mood and Feelings Questionnaire total score.

### 2.2 Multiple regression models

A series of multiple linear regressions were conducted to test the hypothesis that each process variable would predict prospective social anxiety (Hypothesis 1). In each regression model, age, gender, and baseline social anxiety were entered in the first step. One of the process variables was entered in the second step. Table 3 indicates that negative social cognitions (β = 0.22, *p* < .001), safety behaviours (β = 0.24, *p* < .001), self-focused attention (β = 0.12, *p* < .001), and post-event processing (β = 0.10, *p* < .01) significantly predict T2 social anxiety, after controlling for Step One variables. Pre-event processing did not predict T2 social anxiety (β = 0.03, *p* = .40) and was excluded from subsequent regression analysis.

**Table 3:** Results of a series of multiple linear regression analyses predicting T2 social anxiety from process variables, after controlling for age, gender, and T1 social anxiety (*N* = 614).

Variable	Δ*R*^2^	β (SE)	*F*
Negative social cognitions	0.023	0.22 (0.05)	4.21***
Safety behaviours	0.025	0.24 (0.05)	5.25***
Self-focused attention	0.013	0.12 (0.03)	3.55***
Pre-event processing	0.001	0.03 (0.04)	0.84
Post-event processing	0.008	0.10 (0.03)	3.14**

Note. *p < .05. **p < .01. ***p < .001. β *=* standardised beta coefficient; SE = standard error.

A multiple linear regression was conducted to test the hypothesis that each variable would be independently associated with later social anxiety, after controlling for baseline levels of social anxiety (Hypothesis 2). As shown in Table 4, age, gender, and T1 social anxiety entered in the regression model first, explaining 59% of variance in T2 social anxiety. All the process variables were entered in the second step and together they accounted for an additional 4% of variance in T2 social anxiety, *F*(4, 606) = 11.36, *p* < .001. Negative social cognitions (β = 0.13, *p* < .05), safety behaviours (β = 0.16, *p* < .01), and self-focused attention (β = 0.11, *p* < .001) were independent predictors of T2 social anxiety. Post-event processing, however, did not independently predict T2 social anxiety (β = 0.001, *p* = .97). The effects of negative social cognitions, safety behaviours, and self-focused attention on social anxiety remained statistically significant when post-event processing was not included as a predictor of social anxiety.

**Table 4:** Results of a multiple regression analysis predicting T2 social anxiety from process variables, after controlling for age, gender, and T1 social anxiety (*N* = 614).

Variable	β (SE)
Step 1: *Controlled variables*	
Constant	0.04 (0.04)
Age	0.01 (0.03)
Gender (boys = 1, girls = 0)	−0.08 (0.06)
T1 social anxiety	0.75 (0.03)***
Step 2: *Psychological process variables*	
Negative social cognitions	0.13 (0.06)*
Safety behaviours	0.16 (0.05)**
Self-focused attention	0.11 (0.03)***
Post-event processing	0.001 (0.04)

Note. *p < .05. **p < .01. ***p < .001. β *=* standardised beta coefficient; SE = standard error.

## 3 Discussion

In the present study we examined whether negative social cognitions, safety behaviours, self-focused attention, and pre- and post-event processing are associated with prospective levels of social anxiety in a community sample of adolescents. We found that four out of five of these process variables were prospectively associated with social anxiety. Furthermore, three of the variables, namely negative social cognitions, safety behaviours, and self-focused attention were independently associated with later social anxiety.

Results of the present study suggest that the same psychological processes that maintain *adult* social anxiety are implicated in the maintenance of *adolescent* social anxiety. The study provides evidence for temporal precedence, with psychological processes at baseline predicting later social anxiety. Consistent with findings of cross-sectional studies (Hodson et al., 2008; Schreiber et al., 2012), negative social cognitions emerged as a strong predictor of prospective social anxiety, indicating that young people with more negative social cognitions tend to experience more social anxiety symptoms over time. This result lends support to cognitive models of social anxiety emphasising the pivotal role of negative thoughts in the maintenance of social anxiety (Clark & Wells, 1995; Hofmann, 2007; Rapee & Heimberg, 1997). As well as negative social cognitions, safety behaviours strongly predicted prospective social anxiety, which suggests young people who use more safety behaviours tend to experience more social anxiety symptoms. This result is in line with findings of cross-sectional studies with adolescents (Ranta, Tuomisto, Kaltiala-Heino, Rantanen, & Marttunen, 2014; Schreiber et al., 2012) and results of experimental studies with adults (e.g. McManus et al., 2008; Plasencia, Alden, & Taylor, 2011). Self-focused attention was also an independent predictor of prospective social anxiety, suggesting that adolescents who focus internally tend to feel more anxious and avoid more social situations over time. This finding is consistent with concurrent correlational findings (e.g. Hodson et al., 2008) and experimental studies (e.g. Vriends, Meral, Bargas-Avila, Stadler, & Bögels, 2017; Woody & Rodriguez, 2000). Post-event processing was associated with prospective social anxiety, but not after accounting for the other process variables. This may be due to random measurement error arising from the use of a single-item measure, or the time interval between the two measurement points being too short to demonstrate effects. It also may be because post-event processing leads to an increase in social anxiety via other processes (Hirsch, Clark, & Mathews, 2006), for example, post-event processing may intensify negative thoughts, thereby increasing the use of safety behaviours, and worsening social anxiety. Contrary to our hypothesis, we did not find evidence of a prospective relationship between pre-event processing and social anxiety. Partially consistent with this, although pre-event processing was found to be associated with concurrent social anxiety in the studies of Hodson et al. (2008) and Schreiber et al. (2012), it did not emerge as an independent predictor of concurrent social anxiety in either study. This null finding may be a methodological issue, as all three studies relied on a single-item measure of pre-event processing. Further studies using a more robust measure would be helpful to test this possibility.

We note that a relatively small amount of the variance in later social anxiety was explained by the process variables (4%), over and above baseline social anxiety, age and gender (59%). Social anxiety at baseline is strongly correlated with social anxiety at follow-up (*r* = 0.77). Furthermore, at baseline, the psychological process variables are all significantly associated with social anxiety. When controlling for baseline social anxiety, the high stability of social anxiety over time and the considerable concurrent correlations between process variables and social anxiety will explain much of the variance in later social anxiety. That is, by controlling for baseline symptoms it is possible that we are masking or underestimating the effect of the psychological processes that underlie its maintenance.

Although not a main focus of the present study, it is interesting to note the finding that older adolescents reported higher levels of social anxiety than their younger peers. This result is in line with existing literature demonstrating a normative increase in social fears in adolescence (Westenberg, Gullone, Bokhorst, Heyne, & King, 2007), perhaps underpinned by the increasing significance and complexity of social relationships during this time (Rudolph, 2008). Compared to boys, we found that girls were more socially anxious and reported higher levels of negative social cognitions, safety behaviours, and pre- and post-event processing. The observed differences align with findings reported elsewhere that women are more likely to develop social anxiety than men (Asher & Aderka, 2018; Asher et al., 2017).

This study has several strengths, including the use of a prospective survey design, the recruitment of a large sample of adolescents, the inclusion of adapted measures for adolescents, and the use of multiple imputations to account for missing data. However, several limitations should also be noted. First, given that the study was undertaken with an unselected sample, the present findings need to be replicated with a clinical sample. However, we note that there is reason to think that social anxiety varies continuously across normative population (Stopa & Clark, 2001). Second, self-report measures are susceptible to common method bias. Using the same measurement method may introduce artifactual covariance between measured variables, and this could inflate the strength of associations between social anxiety and process variables. Although the present study indicates there is no evidence of multi-collinearity, future studies including behavioural, psychophysiological, and other-informant measures for these processes would be valuable. Third, three of the five psychological processes (self-focused attention, pre-event processing, and post-event processing) were measured with a single-item measure. Single-item measures are prone to random measurement errors and may underestimate the associations between social anxiety and process variables. We note that the two psychological processes (pre- and post-event processing) that did not uniquely predict later social anxiety were measured in this way. This methodological constraint, as well as the relatively short time interval between measurement points, may contribute to false negative outcomes and lead to a conclusion that certain processes are not relevant to the maintenance of adolescent social anxiety, when this conclusion cannot be drawn. Future studies with more reliable measures and longer time intervals are warranted. Fourth, whilst prospective studies are supportive of causality, experimental studies that explicitly manipulate the psychological processes are needed to examine causal hypotheses in adolescents. Fifth, a questionnaire measure for social anxiety was used in the study in order to examine continuous associations amongst the variables of interest, however, inclusion of a validated diagnostic tool would be useful in future studies. Finally, we would encourage future studies that include multiple measurement points to better understand the temporal dynamics of adolescent social anxiety and psychological processes.

The present study has found that negative social cognitions, safety behaviours, self-focused attention, and post-event processing are associated with prospective levels of adolescent social anxiety. Our findings suggest that they may be important targets for treatment and provide support for the use of treatments such as Cognitive Therapy, that are designed to reverse these processes, with adolescents (Ingul, Aune, & Nordahl, 2013; Leigh & Clark, 2016; Melfsen et al., 2011).

## CRediT authorship contribution statement

**Kenny Chiu:** Investigation, Project administration, Formal analysis, Software, Writing - original draft. **David M. Clark:** Conceptualization, Methodology, Supervision, Writing - review & editing. **Eleanor Leigh:** Conceptualization, Methodology, Investigation, Project administration, Supervision, Writing - review & editing.

## Declaration of competing interest

DMC is the co-author of the Clark and Wells’ (1995) cognitive model of social anxiety. The authors have no other conflicts of interest.
