# Supplementary material for: Cognitive predictors of adolescent social anxiety
Source: Behav Res Ther. 2021 Feb;137:103801. doi: 10.1016/j.brat.2020.103801 (PMC7846721; doi:10.1016/j.brat.2020.103801)
Supplement: Multimedia component 1 [file mmc1.doc]

**Supplementary Materials**

**Fig. S.1** Participant flow chart

**
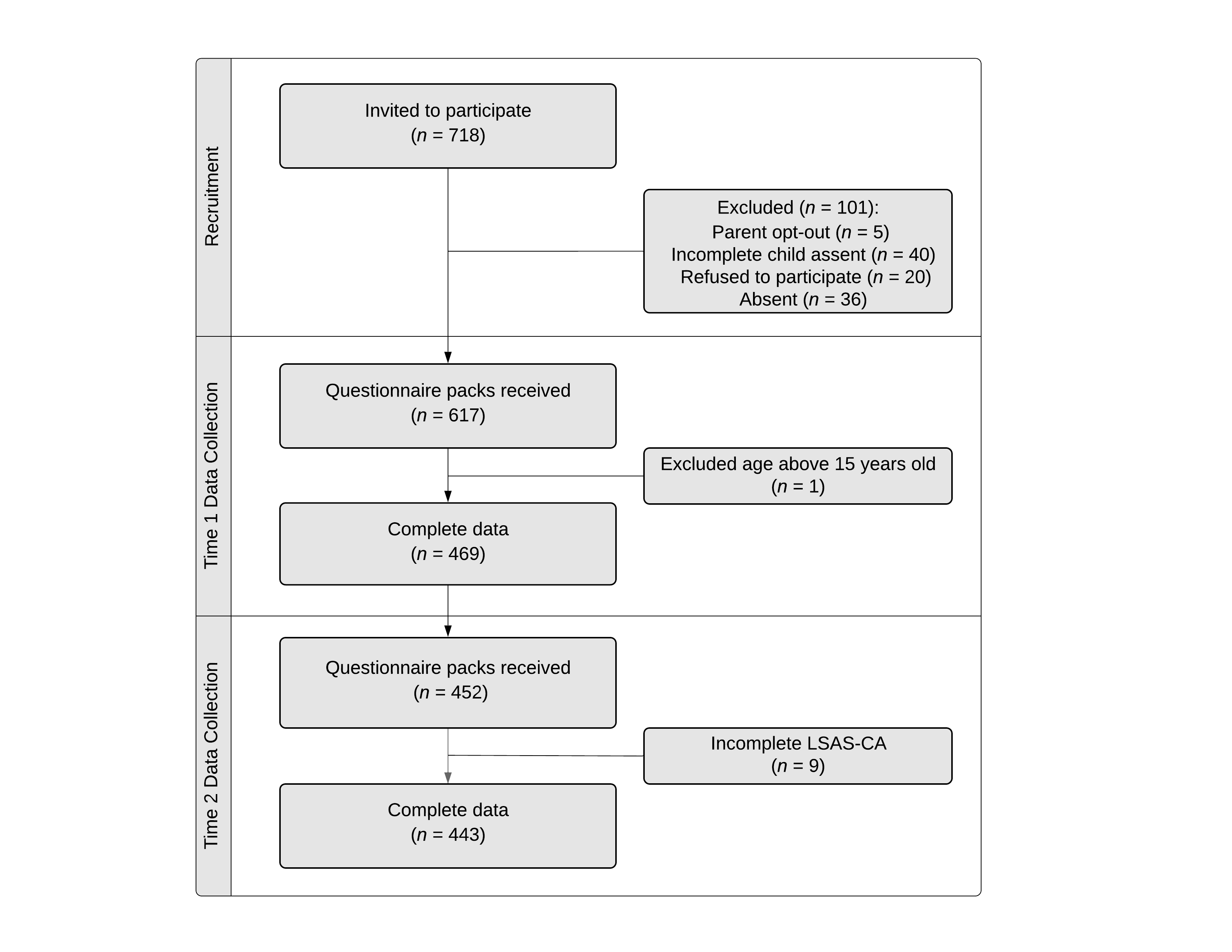
**

**Table S.1** Results of complete case analysis: a series of multiple linear regressions for each process variable predicting T2 social anxiety, controlling for age, gender, and T1 social anxiety (*n* = 351)

| Variable | Δ*R^2^* | β (SE) | *F* |
| --- | --- | --- | --- |
| Negative social cognitions | .002 | 0.23 (0.05) | 24.23*** |
| Safety behaviours | .003 | 0.23 (0.04) | 27.37*** |
| Self-focused attention | .001 | 0.09 (0.03) | 7.48** |
| Pre-event processing | -.002 | 0.03 (0.04) | 0.73 |
| Post-event processing | .001 | 0.09 (0.04) | 6.09* |

*Note. * p* < .05. ** *p* < .01. *** *p* < .001. β *=* standardised beta coefficient; SE = standard error.

**Table S.2** Results of complete case analysis: a multiple linear regression analysis for variable predicting T2 social anxiety, controlling for age, gender, and T1 social anxiety (*n* = 351)

| Variable | β (SE) |
| --- | --- |
| Step 1 |  |
| Constant | 0.06 (0.05) |
| Age | 0.03 (0.03) |
| Gender (boys = 1, girls = 0) | -0.14 (0.07)* |
| Baseline social anxiety | 0.76 (0.04)*** |
| Step 2 |  |
| Negative social cognitions | 0.15 (0.06)* |
| Safety behaviours | 0.15 (0.05)** |
| Self-focused attention | 0.09 (0.03)** |
| Post-event processing | 0.0004 (0.04) |

*Note. * p* < .05. ** *p* < .01. *** *p* < .001. β *=* standardised beta coefficient; SE = standard error.
